# Antiferroptosis therapy alleviated the development of atherosclerosis

**DOI:** 10.1002/mco2.520

**Published:** 2024-04-04

**Authors:** Zhou Yang, Yue He, Dejun Wu, Weihao Shi, Ping Liu, Jinyun Tan, Rui Wang, Bo Yu

**Affiliations:** ^1^ Department of Vascular Surgery Shanghai Pudong Hospital Fudan University Pudong Medical Center Shanghai China; ^2^ Fudan Zhangjiang Institute, Fudan University Shanghai China; ^3^ Shanghai Key Laboratory of Vascular Lesions Regulation and Remodeling Shanghai China; ^4^ Department of Head and Neck Surgery Fudan University Shanghai Cancer Center Shanghai China; ^5^ Shanghai University of Traditional Chinese Medicine Shanghai China; ^6^ Department of Cardiology Shanghai Eighth People's Hospital Shanghai China; ^7^ Shanghai Engineering Research Center of AI Technology for Cardiopulmonary Diseases Shanghai China; ^8^ Department of General Surgery Shanghai Pudong Hospital Fudan University Pudong Medical Center Shanghai China; ^9^ Department of Vascular Surgery Huashan Hospital Affiliated to Fudan University Shanghai China; ^10^ Shanghai University of Traditional Chinese Medicine Department of Cardiology Longhua Hospital Shanghai China; ^11^ Department of Cardiovascular Surgery Nanjing First Hospital Nanjing Medical University Nanjing China

**Keywords:** atherosclerosis, endothelial cells, ferroptosis, glutathione peroxidase 4, oxidized low‐density lipoprotein

## Abstract

Ferroptosis has been confirmed to be associated with various diseases, but the relationship between ferroptosis and atherosclerosis (AS) remains unclear. Our research detailly clarified the roles of ferroptosis in three continuous and main pathological stages of AS respectively (injury of endothelial cells [ECs], adhesion of monocytes, and formation of foam cells). We confirmed that oxidized low‐density lipoprotein (ox‐LDL), the key factor in the pathogenesis of AS, strongly induced ferroptosis in ECs. Inhibition of ferroptosis repressed the adhesion of monocytes to ECs by inhibiting inflammation of ECs. Ferroptosis also participated in the formation of foam cells and lipids by regulating the cholesterol efflux of macrophages. Further research confirmed that ox‐LDL repressedthe activity of glutathione peroxidase 4 (GPX4), the classic lipid peroxide scavenger. Treatment of a high‐fat diet significantly induced ferroptosis in murine aortas and aortic sinuses, which was accompanied by AS lesions and hyperlipidemia. Treatment with ferroptosis inhibitors significantly reduced ferroptosis, hyperlipidemia, and AS lesion development. In conclusion, our research determined that ox‐LDL induced ferroptosis by repressing the activity of GPX4. Antiferroptosis treatment showed promising treatment effects in vivo. Ferroptosis‐associated indexes also showed promising diagnostic potential in AS patients.

## Graphical abstract

Ferroptosis participates in the pathology of atherosclerosis and is a therapeutic target: (1) ox‐LDL induces ferroptosis in endothelial cells. (2) Ferroptosis inhibitors reduce inflammation of endothelial cells and adhesion of monocytes. (3) Ferroptosis regulates the formation of foam cells by regulating cholesterol efflux.

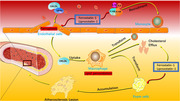

## 1 INTRODUCTION

Atherosclerosis (AS) is the main cause of coronary heart disease, cerebral infarction, and peripheral vascular disease.^1^ Oxidized low‐density lipoprotein (ox‐LDL) plays a key role in the occurrence and development of AS. The ox‐LDL‐induced injury of endothelia1 cells (ECs) is considered to be initial pathogenesis of AS. ox‐LDL promotes the permeability of ECs, causes vacuolar degeneration of the cytoplasm, shrinks the serosal membrane, and eventually leads to cell necrosis.^2^ Moreover, ox‐LDL stimulates the expression of intercellular adhesion molecule‐1 (ICAM‐1) and vascular cell adhesion molecule‐1 (VCAM‐I), which promotes the adhesion and migration of monocytes. Monocytes adhere to the endothelium, migrate into the inner membrane, and finally differentiate into macrophages.^3^ As the key process of AS, macrophages take up large amounts of ox‐LDL to form foam cells. Accumulated foam cell deposition causes the arterial wall to transform from initially having fatty streaks to having more complex fibrous and atheromatous plaques. The outermost macrophage‐derived, rich foam cells of these plaques are prone to causing plaque rupture, leading to thrombosis.^4^

Ferroptosis is an iron‐dependent cell death process that is different from apoptosis, necrosis, and autophagy. The main mechanism underlying ferroptosis is that Fe^2+^ catalyzes the liposome peroxidation of polyunsaturated fatty acids (PUFAs) on the cell membrane, thereby inducing cell death.^5^ The most classic ferroptosis defense pathway involves glutathione peroxidase 4 (GPX4), which specifically catalyzes lipid peroxides to reduce oxidation activity, thus protecting cells from ferroptosis.^6^

Previous studies have found ferroptosis participated in the development of AS, especially injury of ECs.^7^, ^8^ High‐fat diet (HFD) in vivo and ox‐LDL in vitro both induced ferroptosis in ECs by repressing GPX4.^7^ Meanwhile, ferroptosis inhibitors also alleviated AS process in APOE^−/−^ mice.^9^ However, regulation of ferroptosis during whole AS process, especially in macrophages and monocytes, was rarely researched. Meanwhile, the role of ferroptosis in real human samples was also rarely known.

In this research, we performed an in‐depth study of the role of ferroptosis in AS. Starting from ox‐LDL, we innovatively demonstrated the regulation axis of ox‐LDL–GPX4–ferroptosis in AS. Ferroptosis participates in the whole pathology of AS: (1) ox‐LDL induces ferroptosis in endothelial cells (ECs). (2) Ferroptosis inhibitors reduce inflammation of ECs and adhesion of monocytes. (3) Ferroptosis regulates the formation of foam cells by regulating cholesterol efflux. Moreover, our in vivo study revealed the potential of ferroptosis as a therapeutic and diagnostic target, which would be of great interest to the clinical therapy of AS.

## 2 RESULTS

### 2.1 ox‐LDL induced ferroptosis in ECs

**FIGURE 1:**
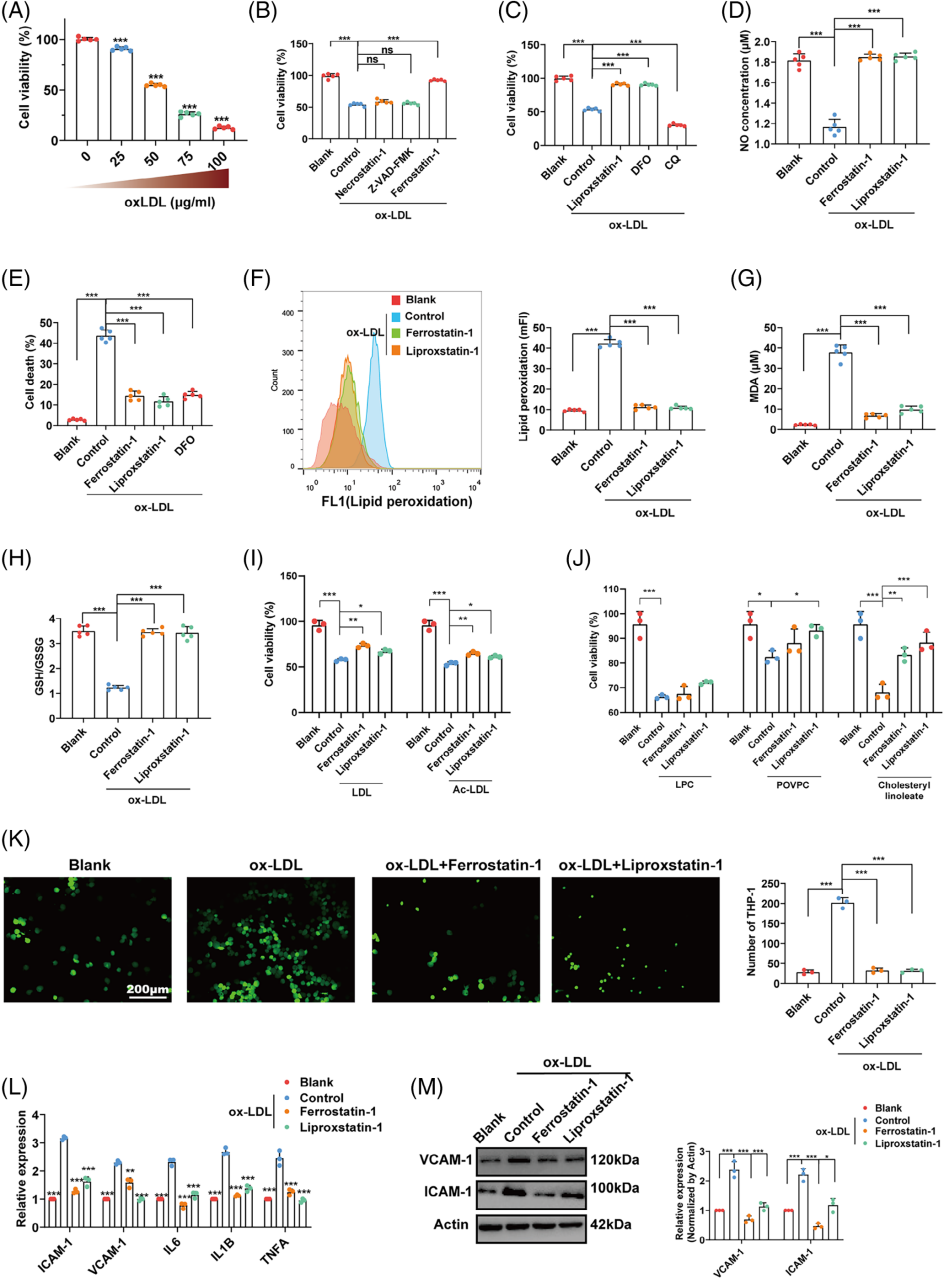
ox‐LDL‐induced ferroptosis in endothelial cells. (A) The viability of HUVECs after treatment with different concentrations of ox‐LDL (0–100 µg/mL, 24 h). (B and C) The viability of HUVECs after cotreatment with ox‐LDL (50 µg/mL) and different inhibitors (necroptosis inhibitor necrostatin‐1: 20 µM, apoptosis inhibitor Z‐VAD‐FMK: 20 µM, ferroptosis inhibitor ferrostatin‐1: 1 µM, ferroptosis inhibitor liproxstatin‐1: 100 nM, ferroptosis inhibitor DFO: 5 µM, and autophagy inhibitor CQ: 5 µM) for 24 h. (D) NO released into the culture medium of HUVECs treated with ox‐LDL with or without ferroptosis inhibitors. (E) Cell death of HUVECs measured by PI staining. (F) Lipid peroxidation levels of HUVECs measured by C11 581/591 BODIPY fluorescent probe (mFI: median fluorescence intensity, detected by flow cytometry). (G) Intracellular malondialdehyde (MDA) levels of HUVECs treated with ox‐LDL with or without the ferroptosis inhibitors ferrostatin‐1 or liproxstatin‐1. (H) Intracellular GSH/GSSG ratio of HUVECs treated with ox‐LDL with or without ferroptosis inhibitor. (I) The viability of HUVECs after cotreatment with LDL/Ac‐LDL (100 µg/mL) and ferroptosis inhibitors for 24 h. (J) The viability of HUVECs after cotreatment with components (lysophosphatidyl choline/LPC, POVPC and cholesteryl linoleate: 50 µg/mL) of ox‐LDL with ferroptosis inhibitors for 24 h. (K) THP‐1 cells adhered to HUVECs after treatment with ox‐LDL and ferroptosis inhibitors for 6 h. (L) The mRNA expression of adhesion factors (ICAM and VCAM) and inflammatory factors (IL6, IL1B, and TNFA) in HUVECs. (M) The protein expression of ICAM‐1 and VCAM‐1 in HUVECs (ns: no significance, *p < 0.05, ***p < 0.001).

First, we confirmed that ox‐LDL repressed the viability of human umbilical vein endothelial cells (HUVECs) in a concentration‐dependent manner. ox‐LDL (25 µg/mL) slightly reduced the viability of HUVECs. When the concentration of ox‐LDL increased to 100 µg/mL, almost all HUVECs were killed (Figure 1A). In the following study, we treated HUVECs with 50 µg/mL ox‐LDL under most conditions unless otherwise specified. To further determine the detailed manner of ox‐LDL‐induced injury in HUVECs, we treated HUVECs with the apoptosis inhibitor Z‐VAD‐FMK, the necroptosis inhibitor Necrostatin‐1, autophagy inhibitor chloroquine (CQ), ferroptosis inhibitors ferrostatin‐1, respectively. CQ enhanced ox‐LDL‐induced injury of EC, indicating autophagy participated in ox‐LDL‐induced injury of HUVECs. Meanwhile, necrostatin‐1 slightly restored ox‐LDL‐induced injury of HUVECs, but not as significant as ferroptosis inhibitors. Therefore, ox‐LDL induces multiple cell death modes. However, ferroptosis inhibitor provides the strongest restore effect, indicating its leading role in ox‐LDL‐induced injury of HUVECs (Figures 1B and C). Similar to the viability assays, ox‐LDL also inhibited nitric oxide (NO) release by HUVECs, an important vasoactive mediator, whereas ferroptosis inhibitors (ferrostatin‐1 or liproxstatin‐1) reversed this inhibition (Figure 1D).

To further confirm that ox‐LDL induces ferroptosis in HUVECs, we investigated a series of ferroptosis‐associated indexes, including cell death (propidium iodide [PI] staining), lipid peroxidation, malondialdehyde (MDA), and GSH/GSSG ratio. As expected, ox‐LDL promoted cell death, the levels of lipid peroxidation, and MDA in HUVECs but repressed GSH/GSSG ratio. Treatment with ferroptosis inhibitors (ferrostatin‐1 or liproxstatin‐1) completely reversed the effect of ox‐LDL (Figures 1E–H). So far, we confirmed that ox‐LDL promoted EC injury by inducing ferroptosis.

Additionally, we also investigated whether natural LDL or acetylated LDL (Ac‐LDL) induced ferroptosis in ECs. Similar to ox‐LDL, the injury induced by LDL and Ac‐LDL could also be reversed by ferroptosis inhibitors (Figure 1I). As ox‐LDL has stronger killing effect at ECs and plays important role in the formation of foam cells, we still focused on ox‐LDL‐induced ferroptosis. Moreover, we also investigated which components of ox‐LDL induced ferroptosis. 1‐Palmitoyl‐2‐(5′‐oxo‐valeroyl)‐sn‐glycero‐3‐phosphocholin (POVPC) and cholesteryl linoleate were confirmed to contribute to ox‐LDL‐induced ferroptosis, but not lysophosphatidyl choline (LPC) (Figure 1J).

### 2.2 Inhibition of ferroptosis reduced adhesion of monocytes

In addition to direct injury, ox‐LDL also promotes the expression of adhesion factors, leading to monocyte adhesion and ECs inflammation. This is another risk factor and pathological process of AS. We found that both ferrostatin‐1 and liproxstatin‐1 significantly repressed the adhesion of THP‐1 cells (monocyte cell line) to HUVECs (Figure 1K). Subsequently, we measured the expression of several key inflammatory factors (TNF‐α, IL6, and IL‐1β) and adhesion factors (ICAM‐1 and VCAM‐1) induced by ox‐LDL in HUVECs. We found that both ferrostatin‐1 and liproxstatin‐1 significantly repressed the expression of these factors in HUVECs (Figures 1L and M).

### 2.3 Inhibition of ferroptosis reduced the formation of foam cells by promoting cholesterol efflux

**FIGURE 2:**
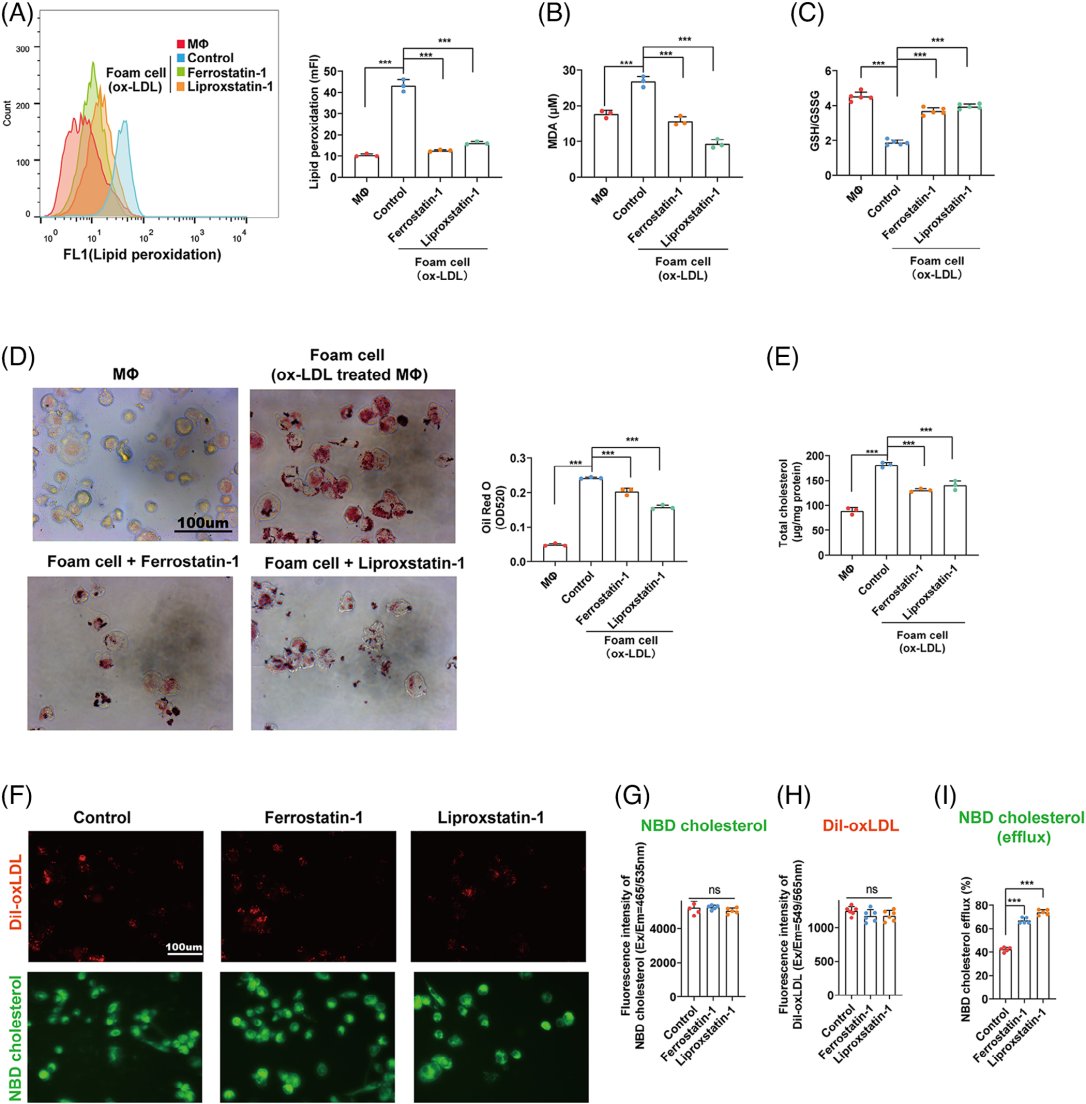
Inhibition of ferroptosis reduced the formation of foam cells by promoting cholesterol efflux. THP‐1 cells were treated with 100 nm PMA for 48 h to induce macrophages, and followed with 50 µg/mL ox‐LDL to induce foam cells for 48 h. Cells were cotreated with the ferroptosis inhibitors ferrostatin‐1 and liproxstatin‐1 and ox‐LDL for 48 h. (A) Lipid peroxidation levels of MΦ and MΦ‐derived foam cells treated with or without ferroptosis inhibitors. (B) Intracellular MDA of MΦ and MΦ‐derived foam cells treated with or without ferroptosis inhibitors. (C) Intracellular GSH/GSSG ratio of MΦ and MΦ‐derived foam cells treated with or without ferroptosis inhibitors. (D) Lipid droplets (LDs) in MΦ and MΦ‐derived foam cells treated with or without ferroptosis inhibitors. After stanning, Oil Red O was eluted by isopropanol and quantified by measuring OD 520. (E) Total cholesterol levels in MΦ and MΦ‐derived foam cells treated with or without ferroptosis inhibitors. (F) Fluorescence images of Dil‐ox‐LDL and NBD‐cholesterol uptake in MΦs. (G) Quantification of Dil‐ox‐LDL uptake measured by flow cytometry. (H) Quantification of NBD‐cholesterol measured by fluorescence intensity. (I) Cholesterol efflux (%) of MΦs treated with or without ferroptosis inhibitors. Stimulated by APOA1 (10 µg/mL, 2 h) (ns: no significance, *p < 0.05, ***p < 0.001).

Recruited monocytes in the previous process (monocytes adhesion to ECs) transforms into macrophages (MΦs), MΦs take up excessive ox‐LDL, resulting in the accumulation of lipids and the formation of foam cells. The formation of foam cells is the core step of AS development.^10^ To investigate whether ferroptosis participated in the formation of foam cells, we treated THP‐1 cells (monocytes cell line) with phorbol‐12‐myristate‐13‐acetate (PMA; 100 nM for 48 h) to induce their differentiation into MΦs, followed by treatment with ox‐LDL (50 µg/mL, 48 h) to further induce foam cell formation. We determined that the levels of lipid peroxidation and MDA were significantly increased and that the GSH/GSSG was significantly decreased in foam cells compared with MΦs. But no cell death was observed in the process of formation of foam cells. Meanwhile, treatment with ferroptosis inhibitors (ferrostatin‐1 or liproxstatin‐1) completely reversed the increase in the levels of lipid peroxidation and MDA and the decrease in the level of GSH/GSSG in foam cells (Figures 2A–C). Subsequently, we investigated lipid droplets (LDs) in foam cells via Oil Red O (ORO) staining. ox‐LDL significantly promoted the accumulation of LDs in MΦs. Treatment with ferroptosis inhibitors successfully eliminated the accumulation of LDs (Figure 2D). Similar to LDs, ox‐LDL promoted the accumulation of total cholesterol (TC) in MΦ/foam cells, whereas ferroptosis inhibitors eliminated this accumulation (Figure 2E). In summary, ox‐LDL induced lipid peroxidation of foam cells (no cell death was observed), and ferroptosis inhibitors repressed the formation of foam cells.

The accumulation of LDs could be caused by changes of two opposite directions: lipid uptake and cholesterol efflux.^11^ We found that ferroptosis inhibitors exerted no effect on the uptake of either ox‐LDL (Dil‐ox‐LDL) or cholesterol (22‐(N‐(7‐Nitrobenz‐2‐Oxa‐1,3‐Diazol‐4‐yl)Amino)−23,24‐Bisnor‐5‐Cholen‐3β‐Ol; NBD‐cholesterol) (Figures 2F–H). However, both ferroptosis inhibitors (ferrostatin‐1 and liproxstatin‐1) promoted cholesterol efflux in ox‐LDL‐treated MΦs (Figure 2I). These results indicated that ferroptosis inhibitors repressed the accumulation of LDs in MΦs, and the formation of foam cells, by promoting cholesterol efflux.

### 2.4 ox‐LDL repressed activity of GPX4

**FIGURE 3:**
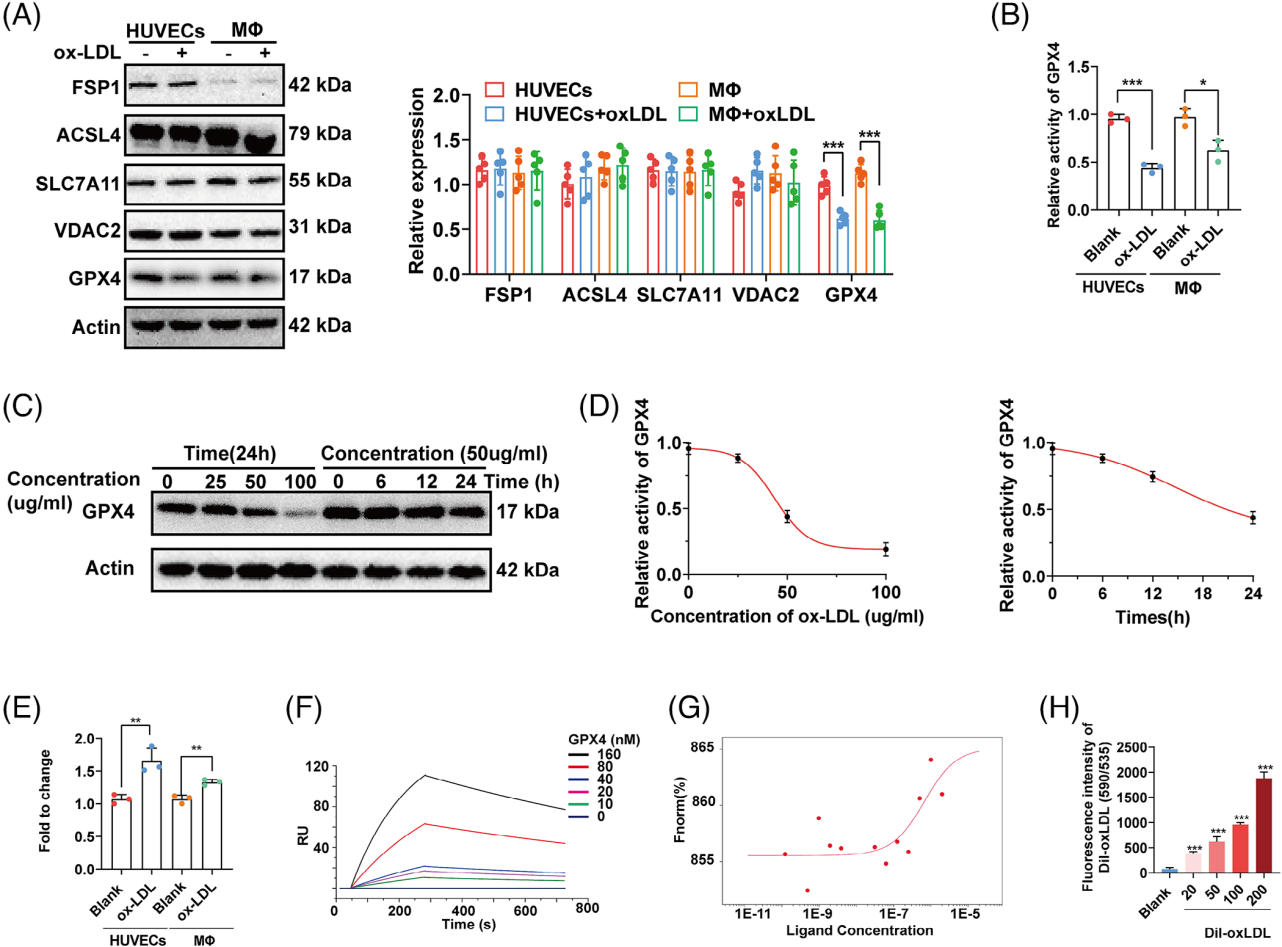
ox‐LDL repressed activity of GPX4. (A) The expression of the main ferroptosis regulators in HUVECs and MΦs with or without ox‐LDL treatment. (B) The activity of GPX4 in HUVECs and MΦs with or without ox‐LDL treatment. (C and D) The expression and activity of GPX4 in HUVECs after treatment with ox‐LDL at different concentrations and times. (E) mRNA expression of GPX4 in HUVECs and MΦs with or without ox‐LDL treatment. (F–H) Interaction of GPX4 and ox‐LDL measured by SPR assay (F), MST assay (G), and Elisa assay (H) (*p < 0.05, **p < 0.01, ***p < 0.001).

To investigate the mechanism underlying ox‐LDL‐induced ferroptosis, the expression of several main ferroptosis regulators (GPX4, ACSL4, SLC7A11, FSP1, and VDAC2) was measured by Western blotting.^7^ We found that only GPX4 expression was repressed by ox‐LDL in both HUVECs and MΦs (Figure 3A). Similar to the trend in expression, ox‐LDL also reduced the enzyme activity of GPX4 in both HUVECs and MΦs (Figure 3B). Subsequently, we determined that ox‐LDL mainly repressed the expression and activity of GPX4 in a concentration‐dependent manner (Figures 3C and D). Interestingly, in contrast to the trend of protein expression, ox‐LDL promoted the mRNA expression of GPX4 (Figure 3E). This indicated that ox‐LDL regulated the GPX4 at the protein level but not at the transcriptional level. The upregulation of GPX4 at the mRNA level seemed to compensate for the reduction in protein levels; therefore, we further suggested that ox‐LDL directly interacted with GPX4. Through SPR and microscale thermophoresis (MST) assays, we confirmed a strong interaction between ox‐LDL and GPX4 (SPR assay: *K*_d_ value: 2.67e−8 M; Figure 3F; MST assay: 6.5853e−7 M; Figure 3G). Similarly in the ELISA assay, the interaction between ox‐LDL and GPX4 quantified by the fluorescence intensity of Dil‐ox‐LDL gradually increased with increasing ox‐LDL concentration (Figure 3H). So far, we had established ox‐LDL interacted with GPX4 and repressed its activity.

### 2.5 Restore of GPX4 alleviates ox‐LDL‐induced endothelial injury and foam cells formation

**FIGURE 4:**
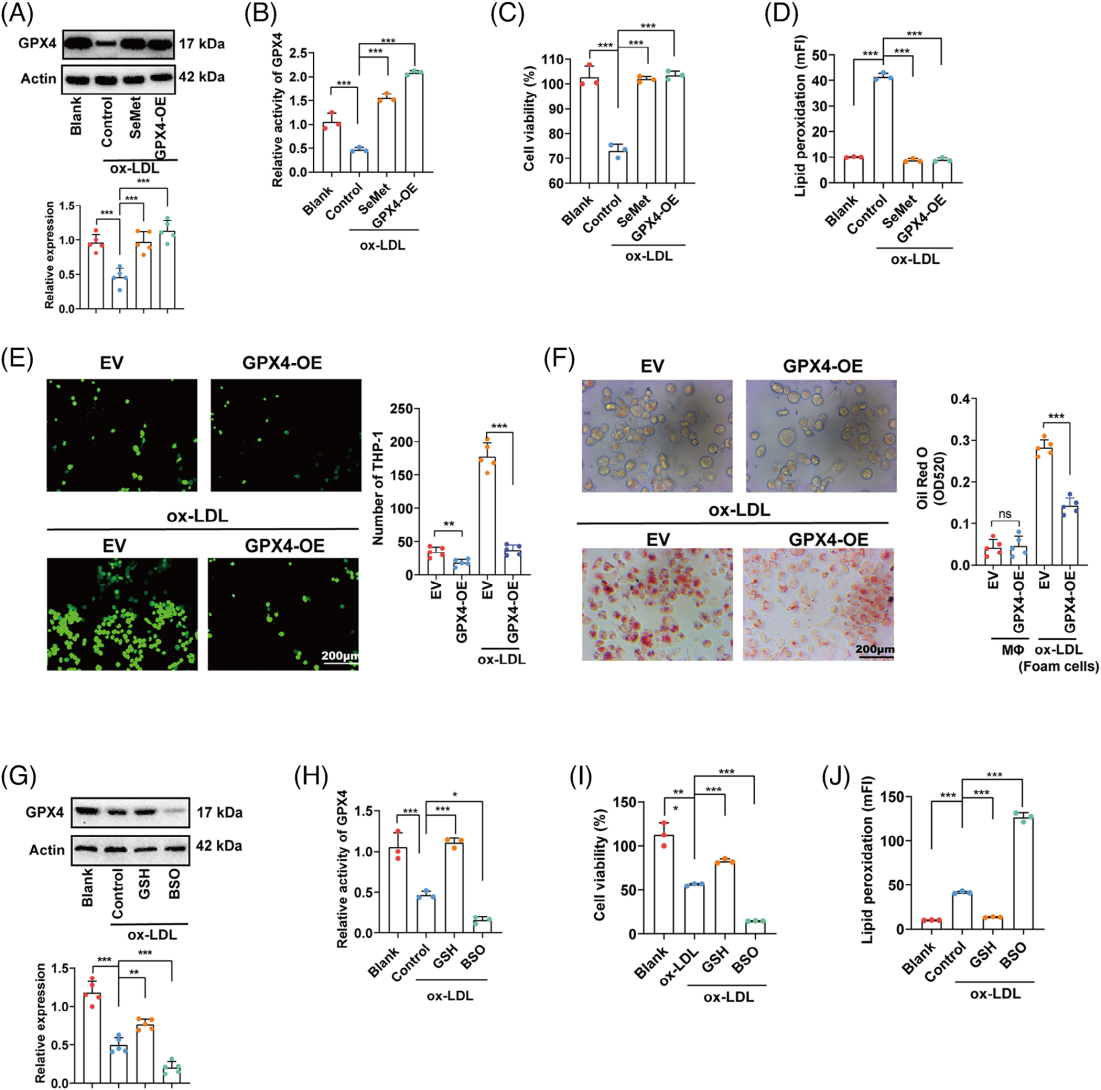
Restore of GPX4 alleviates ox‐LDL‐induced endothelial injury and foam cells formation. (A‐D) The expression of GPX4 (A), the activity of GPX4 (B), cell viability (C), and lipid peroxidation (D) in HUVECs treated with ox‐LDL combined with supplement of SeMet (1 µm, 24 h) or overexpression of GPX4 (GPX4‐OE). (E) THP‐1 cells adhered to HUVECs treated with or without ox‐LDL for 6 h. (F) LDs in MΦ treated with or without ox‐LDL. After stanning, Oil Red O was eluted by isopropanol and quantified by measuring OD 520. (G–J) The expression of GPX4 (G), the activity of GPX4 (H), cell viability (I), and lipid peroxidation (J) in HUVECs treated with ox‐LDL combined with supplement of GSH (1 mM, 24 h) or treatment of BSO (100 µM, 24 h) (*p < 0.05, **p < 0.01, ***p < 0.001).

l‐Selenomethionine (SeMet) supports GPX4 synthesis and activity by providing selenium as cofactor for GPX4.^12^ Both supplement of SeMet and overexpression (OE) of GPX4 restored the expression and activity of GPX4 (Figures 4A and B). As expected, they also successfully rescued cell viability and lipid peroxidation in ox‐LDL‐treated HUVECs (Figures 4C and D). OE of GPX4 in HUVECs also reduced monocytes adhesion; meanwhile, OE of GPX4 in macrophages reduced foam cells formation (Figures 4E and F). As GPX4 catalyzes the reduction of lipid peroxides at the expense of GSH, therefore we further detected the ratio of GSH/GSSG. We have confirmed ox‐LDL repressed the level of GSH/GSSG (Figure 1H); therefore, we further investigated the role of GSH in the ox‐LDL–GPX4 axis. Supplement of GSH restored the activity, but not the expression of GPX4. As expected, supplement of GSH rescued cell viability and lipid peroxidation in ox‐LDL‐treated HUVECs. Oppositely, treatment of γ‐glutamylcysteine synthetase inhibitor l‐buthionine‐(S, R)‐sulfoximine (BSO) further aggravated the repression of both the activity and expression of GPX4. Meanwhile, BSO enhanced repression of cell viability and promotion of lipid peroxidation in ox‐LDL‐treated HUVECs (Figures 4G–J).

### 2.6 Antiferroptosis treatment repressed the development of AS in vivo

**FIGURE 5:**
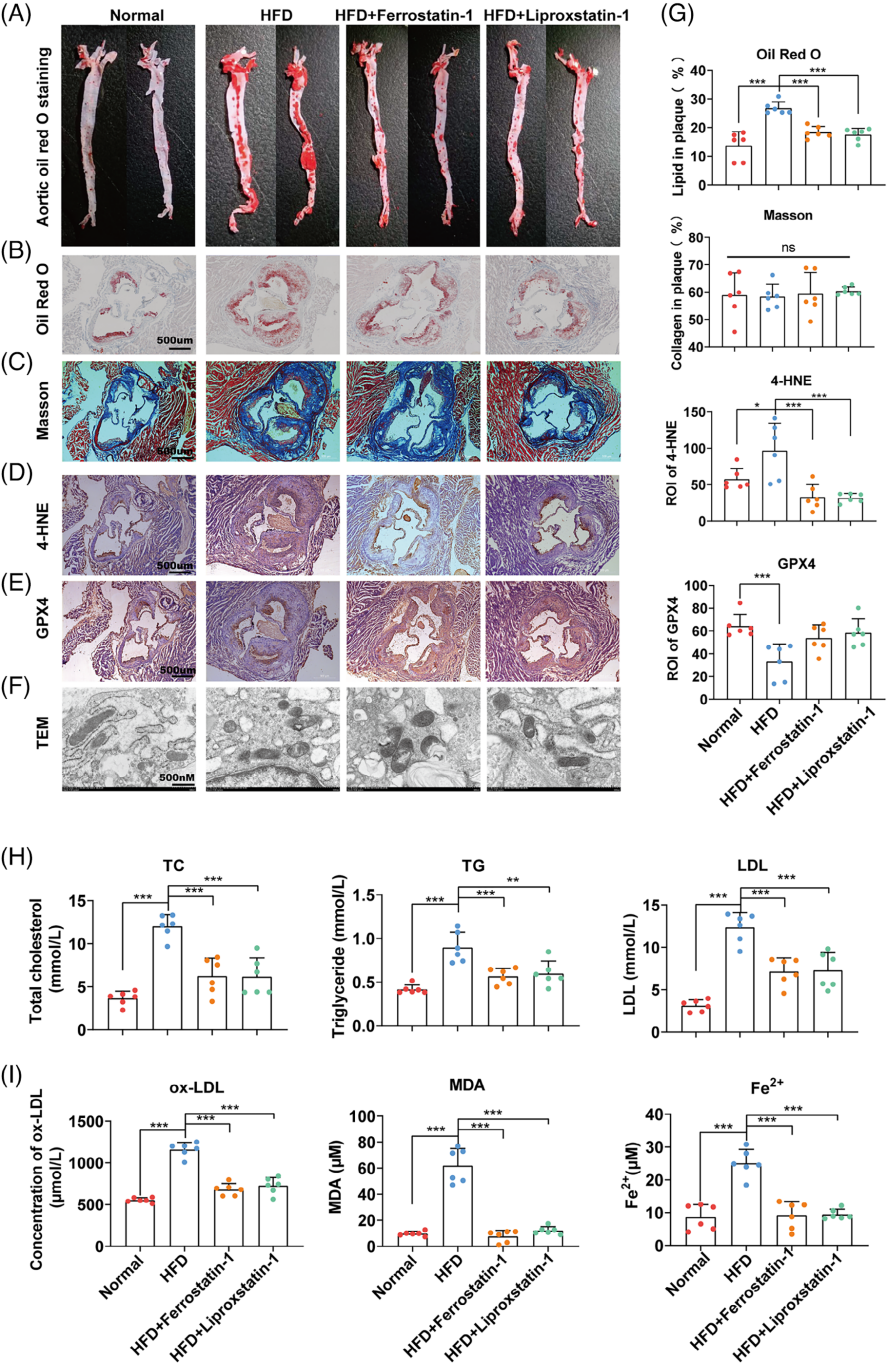
Antiferroptosis treatment repressed the development of AS in vivo. Normal group: normal diet; HFD group: HFD for 3 months; HFD + ferrostatin‐1 group: HFD accompanied by ferrostatin‐1 treatment (1 mg/kg, twice a day, i.p.); HFD + liproxstatin‐1 group: HFD accompanied by liproxstatin‐1 treatment (10 mg/kg, twice a day, i.p.). (A) Aortic AS lesions examined by Oil Red O staining. (B) AS lesions of the aortic sinus examined by Oil Red O staining. (C) Collagen content of the aortic sinus examined by Masson's trichrome staining. (D) IHC of 4‐HEN (ferroptosis marker) in aortic sinus. (E) IHC of GPX4 in aortic sinus. (F) Mitochondrion of aortic sinus observed by TEM. (G) Quantification of B–E. (H) Serum lipid levels (TC, TG, and HDL) of mice in each group. (I) Ferroptosis‐associated indexes (ox‐LDL, MDA, and Fe^2+^) in the serum of each group (*p < 0.05, **p < 0.01, ***p < 0.001).

We have confirmed that ferroptosis plays a key role in ECs injury, adhesion of monocytes, and the formation of foam cells in vitro. To further determine the role of ferroptosis in AS in vivo, we constructed an AS model in APOE−/− mice. After feeding with a HFD for 3 months, the HFD group showed obvious atherosclerotic lesions in the aorta and aortic sinus compared with the normal diet group (normal group). Treating HFD mice with ferrostatin‐1 (1 mg/kg) or liproxstatin‐1 (10 mg/kg) both significantly repressed atherosclerotic lesion formation (Figures 5A, B, and G). We also assessed whether ferroptosis inhibitors regulated the stability of AS by Masson's trichrome staining; however, no change was determined (Figures 5C and G). Furthermore, immunohistochemistry (IHC) of 4‐HNE (a marker for ferroptosis) confirmed that a HFD promoted ferroptosis in the vasculature, whereas ferrostatin‐1 and liproxstatin‐1 repressed ferroptosis (Figures 5D and G). HFD also repressed the expression of GPX4 in the vasculature (Figures 5E and G). In the HFD group, mitochondria shrank, mitochondrial ridge density increased, and mitochondrial membrane ruptured, whereas ferrostatin‐1 and liproxstatin‐1 mitigated these mitochondrial changes (Figures 5F and G). Similarly in the livers of mice, we also confirmed both ferrostatin‐1 and liproxstatin‐1 repressed hepatic lipid accumulation and expression of 4‐HNE (Figure S1A). Moreover, both ferrostatin‐1 and liproxstatin‐1 repressed HFD‐induced upregulation of serum alanine transaminase, aspartate transaminase, gamma glutamyl transpeptidase, nonalcoholic fatty liver disease activity (NAS) score (Figure S1B), indicating their additional therapeutic potential in nonalcoholic fatty liver disease (NAFLD).

Further measurement of a series of blood indexes confirmed that consumption of HFD increased the triglyceride (TG), TC, LDL, ox‐LDL, MDA, and Fe^2+^ levels in mouse serum. Both ferrostatin‐1 and liproxstatin‐1 significantly repressed all these indexes induced by HFD (Figures 5H and I).

### 2.7 Lipidome and gut flora analysis of antiferroptosis‐treated mice

**FIGURE 6:**
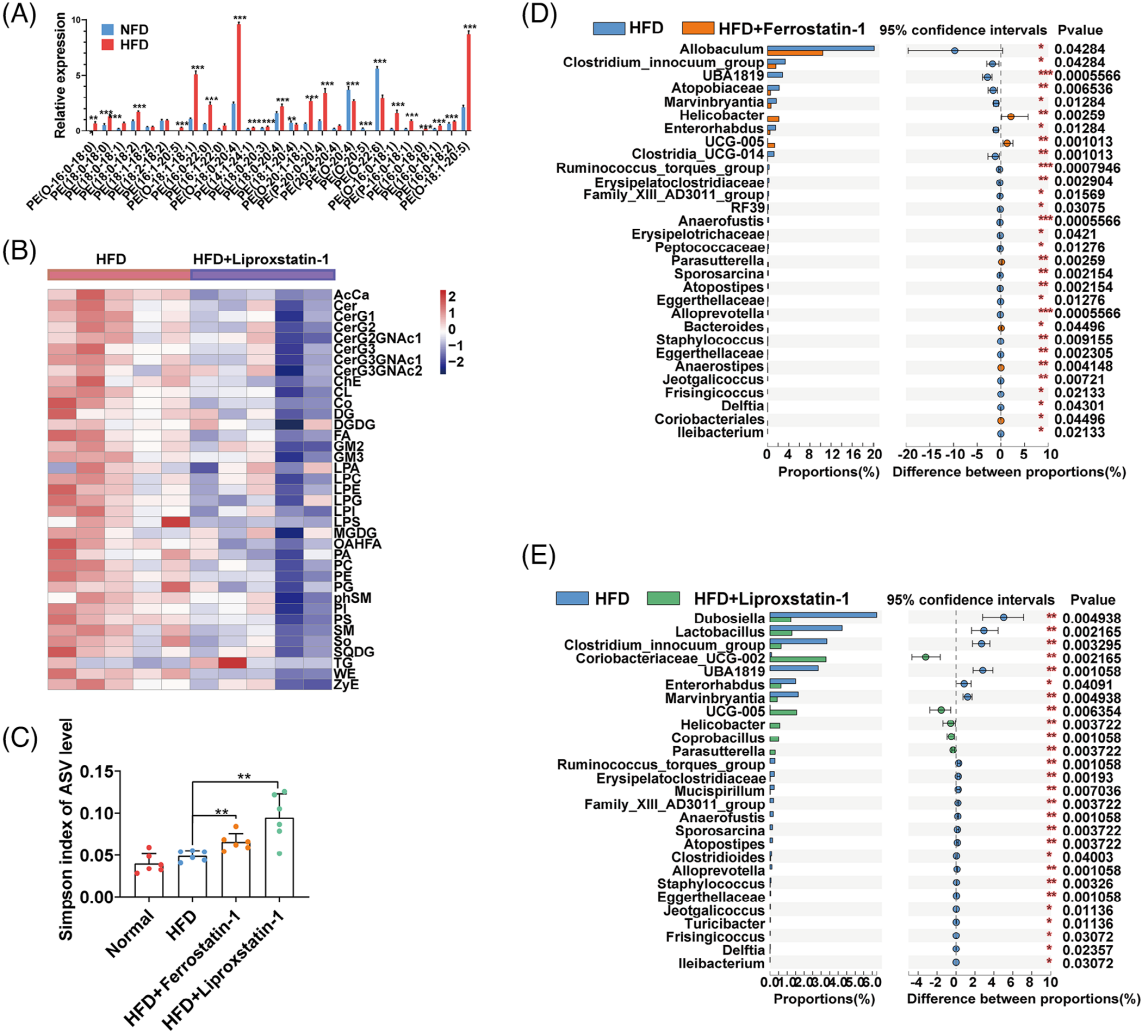
Lipidome and gut flora analysis of ferroptosis‐treated mice. (A) Levels of PUFA‐PEs in normal diet‐fed mice and HFD‐fed mice compared with a reference (Wu Z, etc.). (B) Liproxstatin‐1 repressed various lipid classes in mouse serum. (C) Comparison of the Simpson index of the normal, HFD, ferrostatin‐1, and liproxstatin‐1 groups by Wilcoxon test. (D) Gut microbiota with a significant difference between the HFD group and the ferrostatin‐1 group at the genus level by the Wilcoxon test. (E) Gut microbiota with a significant difference between the HFD group and the liproxstatin‐1 group at the genus level by the Wilcoxon test.

Lipid metabolism shows close association with both ferroptosis and development of AS. Previous research has confirmed that phosphatidyl ethanolamines (PEs) containing PUFAs (especially arachidonic acid (AA, C20:4) and docosahexaenoic acid (DHA; C22:6)) promote lipid peroxidation on the cell membrane, thus leading to ferroptosis.^13^ Therefore, we further performed untargeted lipidomics analysis in the serum of model mice to investigate lipid metabolism regulated by antiferroptosis treatment. Thirty‐seven lipid classes and 1639 lipid species were ultimately identified in the serum of mice (Figures S1C and D). Finally, 586 different lipid species were found to be significantly repressed by liproxstatin‐1. We analyzed previous reported study in the difference between HFD and normal‐fat diet mice. We found HFD feeding significantly promoted the upregulation of various PE‐PUFAs (Figure 6A).^14^ In ourself lipidomics analysis, we further confirmed that liproxstatin‐1 repressed various lipids in serum, such as PE and LPE (Figure 6B). Additionally, almost all PE‐PUFAs were significantly repressed by liproxstatin‐1 (Figure S2).

Imbalance of the gut microbiome also shows close association with AS. To identify the features of the gut microbiome associated with ferrostatin‐1 and liproxstatin‐1 therapy, fecal samples of the four groups were collected for 16S rRNA gene sequencing. Principal coordinates analysis showed that the structure of the gut microbial community was reshaped by HFD feeding (Figure S3A). The Simpson index showed no change in the HFD group compared with the normal group, whereas ferrostatin‐1 and liproxstatin‐1 treatment significantly increased the Simpson index, indicating an increased microbial richness caused by antiferroptosis (Figure 6C). A total of 73 genera were significantly altered between the HFD and normal groups, including diminished numbers of metabolism‐protective microbes such as Lactobacillus, Lachnospiraceae NK4A136 group, and Alloprevotella (Figure S3B). Ferrostatin‐1 alleviated the changes caused by HFD in Allobaculum, UBA1819, Clostridium innocuum group, atopobiaceae, Helicobacter, Clostridia UCG‐014, Ruminococcus torques group, Erysipelatoclostridiaceae, Family XIII AD3011, Marvinbryantia, Anaerofustis, Sporosarcina, Delftia, and Frisingicoccus; however, it enhanced the changes in Atopostipes, Alloprevotella, Erysipelotrichaceae, Staphylococcus, Jeotgalicoccus, and Ileibacterium (Figure 6D). Liprostatin‐1 alleviated the changes in Dubosiella, Bacteroides, Clostridium innocuum group, Clostridioides, UBA1819, Marvinbryantia, Helicobacter, Ruminococcus torques group, Mucispirillum, unclassified f Erysipelatoclostridiaceae, Family XIII AD3011 group, Anaerofustis, Sporosarcina, Frisingicoccus, and Delftia; however, enhanced the changes in Atopostipes, Alloprevotella, Staphylococcus, Turicibacter, Eggerthellaceae, Jeotgalicoccus, Ileibacterium, Lactobacillus, and Coriobacteriaceae UCG‐002 (Figure 6E). Interestingly, the three groups of HFD‐fed mice were mainly associated with adverse changes at the phylum level (Figure S3C). Finally, we also investigated the correlation between gut flora and blood lipids (TG, TC, LDL, and ox‐LDL). We demonstrated Candidatus Saccharimonas, Rikenella, and Desulfovibrio were significantly correlated with low level of TC, TG, LDL, and ox‐LDL (Figure S3D). Overall, we demonstrated ferroptosis inhibitors also regulate gut microbiotas in vivo, whereas specific mechanisms remain to be further elucidated in subsequent experiments.

### 2.8 Ferroptosis‐associated indexes work as potential diagnosis biomarker for AS

**FIGURE 7:**
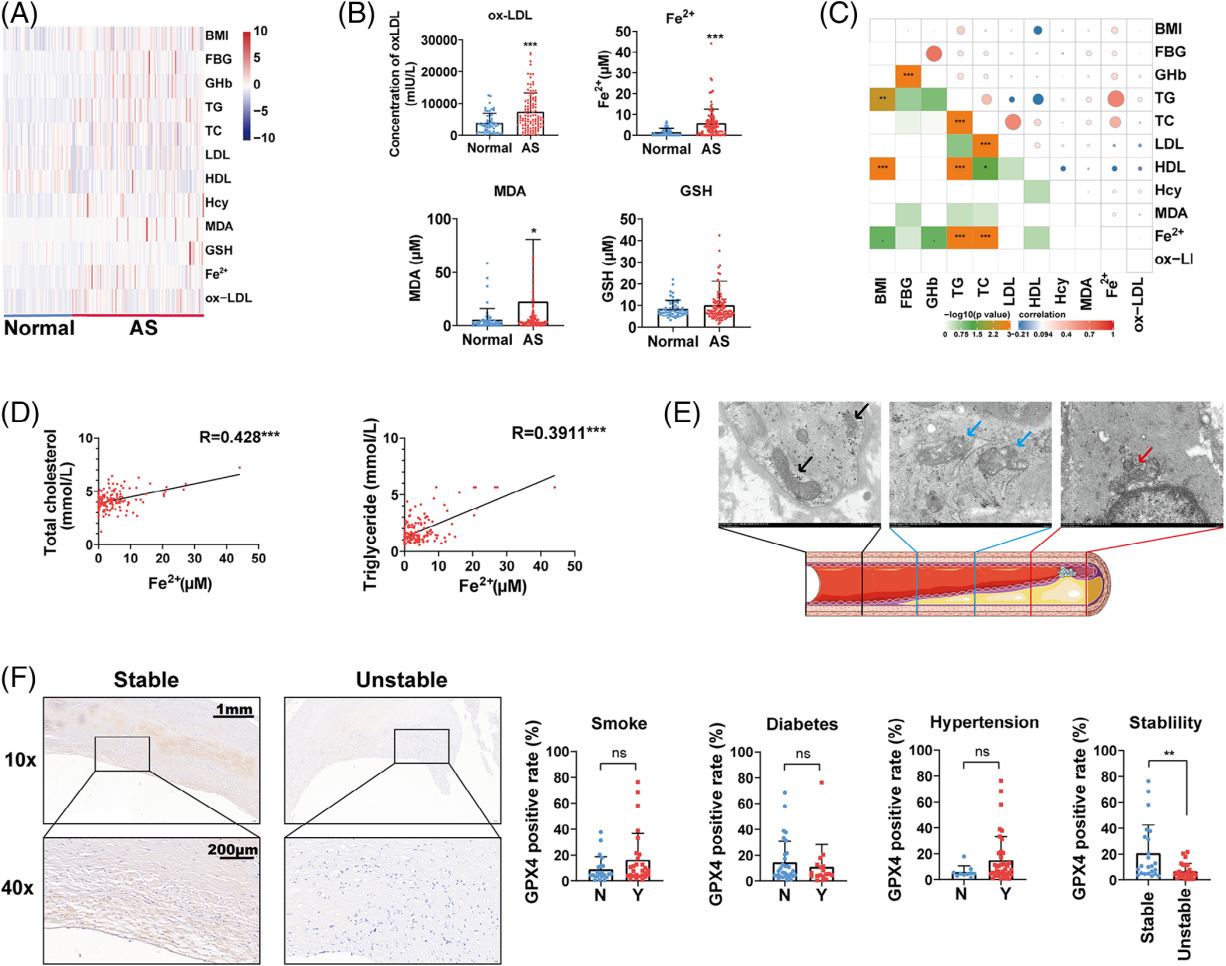
Ferroptosis‐associated indexes work as potential diagnosis biomarker for AS. (A) Heatmap of partial indexes of the community screening population (57 normal people and 111 AS patients). (B) The levels of ox‐LDL, MDA, GSH, and Fe^2+^ in the serum of normal people and AS patients. (C) The correlation between AS‐associated indexes and ferroptosis‐associated indexes analyzed by Pearson analysis. (D) The correlation between Fe^2+^ and TC/TG levels. (E) Mitochondria on different parts of a carotid artery plaque specimen (illustrations used elements from Servier Medical Art: www.servier.fr/servier‐medical‐art). (F) IHC staining of GPX4 in carotid AS plaques (*n* = 50). N: no, Y: yes (*p < 0.05, **p < 0.01, ***p < 0.001).

To explore therapeutic potential of ferroptosis indexes in clinic, we measured four main ferroptosis‐associated indexes (ox‐LDL, MDA, GSH, and nonheme iron/Fe^2+^) in the community screening population, including 57 normal individuals and 111 AS patients diagnosed with carotid plaque. Multiple clinical features and blood indexes were also collected in this analysis (Figure 7A). We found that the ox‐LDL, MDA, and Fe^2+^ levels were all significantly increased in AS patients compared with normal people, whereas the GSH level showed no significant difference (Figure 7B). In further analysis, we found that the ox‐LDL level was increased in the male, smoking, drinking, and AS populations; the Fe^2+^ level was increased in the smoking, drinking, hypertension, overweight (BMI≥24), and AS populations; and the MDA level was increased in the AS population (Table 1). We also analyzed the correlation between lipid indexes and ferroptosis‐associated indexes (Figure 7C). We found that the Fe^2+^ level was significantly correlated with both the TC and TG levels (R: 0.428 and 0.3911, *p* value < 0.001; Figure 7D). We also observed the mitochondria on different parts of a carotid artery plaque specimen derived from carotid endarterectomy (CEA) via transmission electron microscope (TEM). Upon approaching the core of the plaque, mitochondria become smaller, mitochondrial ridges were reduced, and mitochondrial outer membranes were broken (Figure 7E). Finally, we detected expression of GPX4 in 50 cases of carotid AS plaques. GPX4 was significantly downregulated in unstable carotid plaques compared with stable carotid plaques, which indicated GPX4 may be associated with the stability of carotid plaques (Figure 7F).

**TABLE 1:** The association between ferroptosis indexes and clinical features in community screening population.

		ox‐LDL (mIU/L)		Fe^2+^ (µM)		MDA (µM)	
Characteristics	Number	Mean ± SD	*p* Value	Mean ± SD	*p* Value	Mean ± SD	*p* Value
Age							
<60	53	6360 ± 695		3.32 ± 4.75		15.66 ± 58.8	
≥60	115	6042 ± 514	0.722	4.65 ± 6.37	0.177	16.98 ± 42.9	0.884
Gender							
Male	75	7646 ± 5306		4.79 ± 7.02		16.1 ± 57.9	
Female	93	4928 ± 5123	0.001^***^	3.78 ± 4.85	0.274	16.9 ± 39.2	0.922
Smoke							
N	126	5473 ± 5189		3.53 ± 4.58		16.34 ± 41.67	
Y	42	8146 ± 5440	0.007^**^	6.35 ± 8.55	0.007^**^	17.23 ± 65.03	0.918
Drink^b^							
N	136	5776 ± 5227		3.72 ± 5.15		18.73 ± 53.35	
Y	28	8212 ± 5888	0.03^a^	5.26 ± 4.72	0.145	7.25 ± 9.87	0.021^a^
Sport							
N	108	6369 ± 5266		4.36 ± 6.16		19.12 ± 56.91	
Y	60	5731 ± 5556	0.469	4 ± 5.52	0.703	11.96 ± 26.67	0.36
Stroke family history							
N	146	5953 ± 5351		3.98 ± 5.97		17.34 ± 51.51	
Y	19	7508 ± 4992	0.235	5.65 ± 5.38	0.247	10.15 ± 16.25	0.547
Hypertension							
N	109	5460 ± 4730		3.31 ± 5.71		14.31 ± 49.13	
Y	59	7400 ± 6220	0.039^*a^	5.92 ± 5.99	0.007^**^	20.72 ± 47.01	0.408
Dyslipidemia							
N	152	6000 ± 5416		4.25 ± 6.11		16.38 ± 48.8	
Y	16	7482 ± 4784	0.295	4.04 ± 3.82	0.892	18.33 ± 45.34	0.873
Diabetes							
N	151	5936 ± 5115		3.85 ± 5.5		15.57 ± 47.33	
Y	17	7968 ± 7148	0.269	7.59 ± 8.35	0.013^*a^	27.34 ± 57.51	0.431
BMI							
<24	60	6112 ± 5695		5.17 ± 6.66		9.35 ± 26.56	
≥24	108	6159 ± 5198	0.957	2.53 ± 3.79	0.001^***^	20.57 ± 56.7	0.084
Carotid plaque							
N	57	3800 ± 3041		1.48 ± 1.73		5.34 ± 10.64	
Y	111	7344 ± 5888	0.000^***^	5.65 ± 6.78	0.000^***^	22.32 ± 58.25	0.004^**^

^a^ Wilcoxon rank sum test was applied. *p < 0.05, **p < 0.01, ***p < 0.001

^b^ Data of part patients were not acquired.

## 3 DISCUSSION

Previous research has confirmed that the ferroptosis marker prostaglandin‐endoperoxide synthase 2 is overexpressed in human coronary artery AS.^15^ Additionally, ferroptosis inhibitors (ferrostatin‐1) alleviate AS by attenuating lipid peroxidation and endothelial dysfunction.^7^ These studies indicated that ferroptosis plays a key role in AS, but the specific mechanism remains to be explored.

ox‐LDL, the key factor of AS pathogenesis, is abundant in PUFAs. PUFA is an important catalyst for ferroptosis.^16^ Thus, we must consider whether ox‐LDL can directly induce ferroptosis. As the initial pathological process of AS, ox‐LDL‐induced injury of ECs was confirmed to be significantly rescued by ferroptosis inhibitors. Further research also confirmed that ox‐LDL promoted the lipid peroxidation and MDA levels but reduced the GSH level; moreover, ferroptosis inhibitors rescued these changes. All these results indicated that ox‐LDL directly induced ferroptosis in ECs, which is consistent with previous reports.^9^ Furthermore, we also found that not only ox‐LDL but also natural LDL and Ac‐LDL induced ferroptosis. Additionally, we determined that cholesteryl linoleate, the main PUFA located in the hydrophobic core of all LDL modification types, contributed to ferroptosis. Another oxidative phospholipid POVPC located only in ox‐LDL also contributed to ferroptosis.^17^ In summary, based on previous reports, we further found different types of LDL modifications could induce ferroptosis via cholesteryl linoleate. Additionally, we also confirmed that a ferroptosis inhibitor alleviated the ox‐LDL‐induced inflammation of ECs and repressed the adhesion of monocytes. This is the next pathological process of AS after EC injury.^18^

Previous studies have found excessive iron might regulate the formation of foam cells, as well as polarization of macrophages.^19^, ^20^ Meanwhile, iron metabolism also regulates the inflammation of macrophages.^21^ However, a direct association between ferroptosis and macrophages/foam cells was not established. Therefore, we assessed the role of ferroptosis in the third pathological process of AS, as well as the key process, namely, the formation of foam cells. Similar to ECs, ox‐LDL‐treated macrophages/foam cells showed increased lipid peroxidation and MDA levels but decreased GSH levels. Moreover, ferroptosis inhibitors reversed these changes. We suggest that ox‐LDL significantly promotes lipid peroxidation in macrophages and places them on the edge of ferroptosis. This feature was just the basis for the formation of foam cells.^22^ The formation of foam cells depends on the combined effects of lipid absorption, cholesterol esterification, and cholesterol efflux.^4^ We confirmed that ferroptosis inhibitors reduced the formation of foam cells by regulating cholesterol efflux from macrophages.

To further determine the mechanisms underlying ox‐LDL‐induced ferroptosis and lipid peroxidation, we measured the expression of several main ferroptosis regulators reported before, including GPX4, FSP1, ACSL4, SLC7A11, and VDAC2.^23^, ^24^, ^25^ Only GPX4 expression, as well as activity, were significantly inhibited by ox‐LDL in both HUVECs and macrophages. Interestingly, the mRNA expression of GPX4 was upregulated by ox‐LDL, which indicated that ox‐LDL repressed GPX4 at the protein level. Subsequently, we confirmed that ox‐LDL directly interacted with the GPX4 protein through Elisa, SPR, and MST assays, respectively. We successfully determined ox‐LDL interacted with GPX4, thus repressing its activity. As we know, GPX4 is also a selenoprotein and contains the rare amino acid selenocysteine in its active site.^26^ As expected, supplementation with selenium or OE of GPX4 rescued activity of GXP4 and ox‐LDL‐induced ferroptosis in HUVECs. GPX4 catalyzes the reduction of lipid peroxides and PUFAs at the expense of reduced GSH, thus protecting cells from ferroptosis. We also confirmed supplement of GSH rescued activity of GXP4 and ox‐LDL‐induced ferroptosis in HUVECs, whereas inhibition of GSH aggravated ox‐LDL repressed of GPX4 activity and ox‐LDL‐induced ferroptosis. Previous studies have identified several classes of ferroptosis inducers (FINs). Class 1 FINs (Erastin) inhibit SLC7A11‐mediated cystine transport and consume GSH. Class 2 FINs (RSL3) inhibit GPX4 activity by directly binding. Class 3 FINs (FIN56) induce ferroptosis by depleting both GPX4 protein and coenzyme Q10.^27^, ^28^ ox‐LDL directly bound to GPX4, repressed its expression and activity, consumed GSH, and finally induced ferroptosis. It makes ox‐LDL a mixture contains features of all three classes of FINs.

We further established an AS model in APOE^−/−^ mice to assess the effect of antiferroptosis treatment in vivo. Consumption of a HFD not only promoted hyperlipidemia (increase in ox‐LDL, TC, TG, and HDL level) and AS lesion formation in mice but also induced ferroptosis in the vessels (OE of 4‐HNE and breakdown of mitochondria) and an increase in serum ferroptosis‐associated indexes (MDA and Fe^2+^). As expected, treatment with ferrostatin‐1 and liproxstatin‐1 significantly alleviated ferroptosis features, hyperlipidemia, and AS lesions in mice. Subsequently, we further performed serum lipidomic analysis to assess the effect of antiferroptosis treatment in metabolism and microbiology. Antiferroptosis treatment significantly alleviated dyslipidemia induced by HFD, which has been confirmed to be a crucial risk factor for the development of AS.^29^ Further lipidomic analysis confirmed antiferroptosis repressed various lipids in serum. Among them, PE‐PUFAs were reported as the ringleader for ferroptosis. Combined with previous research, which indicated PE‐PUFAs in serum were overexpressed by HFD, we demonstrated antiferroptosis treatment rescued PE‐PUFAs increased by HFD in serum. Additionally, gut microbiota has been increasingly recognized as an important regulator of glucose and lipid metabolism in AS and other metabolic syndromes. In our study, 12 weeks of HFD feeding deteriorated the gut homeostasis, while key microbial representative of three HFD‐treated group showed an obvious difference, indicating that antiferroptosis treatment not only improved dyslipidemia in HFD fed mice but may also by regulating gut microbiota. According to LEfSe analysis, antiferroptosis effectively increased the abundance of short‐chain fatty acids (SCFA) producing bacteria genera, such as Blautia, Romboutsia, Lactococcus, Clostridium sensu stricto 1, Anaerostipes, Bifidobacterium in ferrostatin‐1 group and Allobaculum, Faecalibaculum, Erysipelatoclostridium, UCG‐005, Akkermansia, Eubacterium nodatum, Robinsoniella, Parasutterella, Paludicola, and Christensenellaceae R‐7 in liproxstatin‐1 group.^30^, ^31^ SCFAs are anti‐inflammatory and antiobesity metabolites of gut flora, which display a great influence on gut homeostasis, suggesting that antiferroptosis may improve dyslipidemia by regulating gut flora, especially SCFA producing gut flora.^32^ These sequencing data provided further insight into antiferroptosis to regulate the metabolic disorder through the gut flora. Compared with a single pathway, perhaps the dual regulation of ferroptosis and gut microflora is a more attractive strategy for the treatment of metabolic diseases. Further exploration of the Spearman correlation analysis showed that some specific genera, such as Candidatus Saccharimonas, Rikenella, and Desulfovibrio, strongly associated low levels of all four blood lipid parameters, indicating these genera may serve as a potential prebiotic agent in the prevention of dyslipidemia.^33^ As a “star target” for the treatment of metabolic diseases, whether these gut microbiotas can regulate dyslipidemia and insulin resistance remains to be further elucidated in subsequent experiments.

Gut microbiota metabolites constitute a crucial component of the gut–liver axis. Previous studies have demonstrated that dysbiosis of the gut microbiome could impact the development of NAFLD by promoting endogenous ethanol production, altering bile acid composition, and reducing the synthesis of SCFAs and butyrate.^34^, ^35^ In this study, ferroptosis inhibitors significantly increased the Simpson index compared with the HFD group, indicating an increased microbial richness caused by antiferroptosis. Additionally, probiotics such as bifidobacterium, which could improve metabolic disorders by regulating lipid metabolism and producing SCFAs, were significantly increased by ferroptosis inhibitors, hinting an antiobesity potential of ferroptosis inhibitors.^36^ Notably, ferroptosis was significantly correlated with the NAS. However, due to the limitation of our study, specific mechanisms remain to be further elucidated in subsequent experiments.

Finally, we further investigated the levels of ferroptosis‐associated indexes in clinical AS patients. Compared with normal people, the serum MDA and Fe^2+^ levels were significantly increased in AS patients. Furthermore, the MDA and Fe^2+^ levels were also closely related to various risk factors for AS, including smoking, drinking, overweight, diabetes, hypertension, and so on. These factors were all reported to contribute to the development of AS.^29^ It also suggested that ferroptosis may also have an important regulatory role in other diseases related to these factors. For insistence, ferroptosis has been confirmed to be related to diabetes and obesity.^37^, ^38^

Copper is an essential metallic element in the human body, serving as a cofactor for enzymes involved in mitochondrial respiration, oxidation‐reduction reactions, and biomolecule synthesis. Previous reports have reported copper participates in the crosstalk of ferroptosis and metabolic disease. Serum copper bioavailability has been confirmed to be associated with the risk of cardiovascular events in obese patients with hepatic steatosis. Notably, serum copper showed negative correlation with intima–media thickness, a noninvasive diagnostic tool for premature or subclinical AS identification, as well as functional and structural marker of this process.^39^ In another study, bathocuproinedisulfonic treatment caused copper depletion, leading to depolarization of the mitochondrial membrane potential, increased levels of reactive oxygen species (ROS), oxidative stress induction, and decreased glutathione levels. These effects are similar to those of ox‐LDL.^40^ These studies suggested a complex role of copper in the crosslink of ferroptosis and AS.

In conclusion, our research not only demonstrated the mechanism of ox‐LDL‐induced ferroptosis but also disclosed the therapeutic potential of antiferroptosis treatment and the diagnosis potential of ferroptosis indexes in AS.

## 4 MATERIAL AND METHODS

### 4.1 Cell culture, transfection, and reagents

HUVECs were acquired from ScienCell Research Laboratories (Carlsbad, CA, USA) and cultivated in endothelial cell medium (ECM) containing 5% FBS and 1% endothelial cell growth supplements (ScienCell Research Laboratories). Human myeloid leukemia mononuclear cell line THP‐1 was purchased from the University of Colorado Cancer Center Cell Bank and cultured in RPMI‐1640 medium (Invitrogen, Carlsbad, CA, USA), supplemented with 10% FBS (Invitrogen) at 37°C in a 5% CO_2_ atmosphere.

LDL, ox‐LDL, Ac‐LDL, and Dil‐ox‐LDL were purchased from Peking Union‐Biololgy Co. Ltd. (Beijing, China). The colorimetric determination of TBARS (thiobarbituric acid [TBA] reactive substances) involves the use of MDA as a standard. The concentration of MDA is measured at 12.0 nM per mg of protein in ox‐LDL. Recombinant human GPX4 was purchased from Abcam (Cambridge, UK). Necrostatin‐1 (HY‐15760), carbobenzoxy‐valyl‐alanyl‐aspartyl‐[O‐methyl]‐fluoromethylketone (Z‐VAD‐FMK, HY‐16658B), ferrostatin‐1 (HY‐100579), liproxstatin‐1 (HY‐12726), deferoxamine (DFO; HY‐B1625), and CQ (HY‐17589A) were all purchased from MedChemExpress (Monmouth Junction, NJ, USA). POVPC (Cat. 870606P), LPC (Cat. 1372050), and cholesteryl linoleate (Cat. C0289) were purchased from Sigma–Aldrich Corp. (St. Louis, MO, USA). NBD‐cholesterol (Cat. N1148) was purchased from ThermoFisher Corp. (MA, USA).

### 4.2 Measurement of cell viability, cell death, and lipid peroxidation

10^4^ cells suspended in 100 µL medium were seeded into 96‐well plates overnight. After treatment of different reagents for 24 h (ox‐LDL, etc.), the cell viability was measured by 10 µL CCK8 reagent (Dojindo Molecular Technologies, Kumamoto, Japan) per well. After incubating for 2 h, the absorbance of the cells was measured at a wavelength of 450 nm (OD450).

To assess lipid peroxidation and cell death, a total of 10^6^ cells were placed in 2 mL of medium and seeded into six‐well plates overnight. Following treatment with various reagents for an additional 24 h, the cells were exposed to either 2 µM C11 BODIPY 581/591 from Invitrogen to detect lipid peroxidation or 1 µg/mL PI from Roche in PBS to detect cell death. After being washed twice with PBS, the cells were collected and then analyzed using fluorescence activated cell sorting with FL1 for C11 BODIPY 581/591 and FL2 for PI.

### 4.3 Measurement of GSH/GSSG, Fe^2+^, MDA, and activity of GPX4

All these assays were performed according to the manufacturer's instructions. MDA Assay Kit (Cat. S0131; Beyotime, Shanghai, China) was based on the reaction of MDA and TBA. GSH assays kit (Cat. S0053; Beyotime) was based on the reaction of GSH and 5,5′‐dithiobis‐2‐nitrobenoic acid reacts to produce 2‐nitro‐5‐mercaptobenzoic acid and glutathione disulfide (GSSG). Iron Assay Kit (Cat. ab83366; Abcam) was based on the reaction of Fe^2+^ and Ferene S to produce a stable‐colored complex.

Activity of GPX4 was detected as previous report.^41^ Briefly, 10^7^ cells were lysed with 500 µL lysis buffer (0.1 M KH_2_PO_4_/K_2_HPO_4_, 0.15 M KCl, 0.05% (w/v) CHAPS). Then, lysate was added with 180 IU/mL glutathione reductase (GR), 3 mM NADPH, and 0.025 mM P‐COOH to trigger GPX4 enzyme activity. The activity of GPX4 was calculated by detecting the decrease in NADPH (OD340). GR and NADPH were included in commercial assay kit (S0056; Beyotime), P‐COOH was inhouse made following previous report using phosphatidylcholine (Cat. P3782; Sigma–Aldrich) and soybean lipoxidase type IV (Cat. L6632; Sigma–Aldrich).^42^ Briefly, 250,000 U soybean lipoxidase type IV and 5 mg phosphatidylcholine were added in 20 mL Tris/Base buffer (0.2 M, pH 8.8, containing 3 mM sodium deoxycholate) and reacted at room temperature for 1 h. Then, PCOOH was separated from Tris/Base buffer using Sep‐Pak C18 cartridge (Waters; WAT 022515).

### 4.4 Induce of foam cells and Oil Red O staining

10^6^ THP‐1 cells seeded in six‐well plate were treated with 100 nM PMA (Sigma–Aldrich) for 48 h to induce differentiation into macrophages. Then, induced macrophages were further treated with 50 µg/mL ox‐LDL for 24 h to induce foam cells.

For Oil Red O staining, cells were fixed with 4% formaldehyde for 15 min and then stained with filtered Oil Red O (Cat. O0625; Sigma–Aldrich) for 30 min at room temperature. After rinsing with 60% isopropanol, pictures of cells were captured with a microscope. Subsequently, the Oil Red O was extracted with isopropanol and quantified at OD510.

### 4.5 Cell adhesion assay

HUVECs were stimulated with 50 µg/mL ox‐LDL and incubated with CMFDA (Invitrogen)‐labeled THP‐1 monocytes at 37°C for 30 min. Then, cells were washed twice with PBS, and adherent THP‐1 was captured by microscope.

### 4.6 Uptake of Dik‐ox‐LDL and NBD‐cholesterol

The macrophages were incubated with Dil‐ox‐LDL (50 µg/mL) or NBD‐cholesterol (0.5 mg/mL; Sigma–Aldrich) for 6 h at 37°C. Fluorescence microscope and flow cytometer were used to capture pictures and detect fluorescence intensity, respectively.

### 4.7 Measurement of TC and cholesterol efflux

The macrophages were incubated with NBD‐cholesterol (0.5 mg/mL) for 6 h. After cholesterol loading, the cells were washed, equilibrated for 2 h, and then incubated with APOA1 (10 µg/mL; PeproTech Inc., Rocky Hill, NJ, USA) for 2 h. Control wells were treated with only 0.2% BSA to measure background. The culture medium was collected, and the cells were lysed with 0.3 M NaOH at 37°C for 15 min. The fluorescence intensity of the cell lysate and culture medium were both measured with a microplate spectrophotometer. The calculation of efflux rate is as follows: efflux rate (%) = medium count/(medium count + cell lysate count) × 100.

### 4.8 Western blotting analysis and quantitative PCR

Western blotting and quantitative PCR (RT‐qPCR) were performed following the previous report.^43^ Following antibodies were used in Western blotting: Actin, FSP1, ACSL4, SLC7A11, VDAC2, and GPX4 rabbit polyclonal antibodies (1:1000; Abcam); VCAM‐1 and ICAM‐1 (1:500; Santa Cruz Biotechnology, Inc, Dallas, TX, USA). The primers used in RT‐qPCR are shown in Table S1.

### 4.9 In vitro ox‐LDL–GPX4 interaction analysis

For ELISA assay, rhGPX4 (10 µg) was absorbed into a 96‐well black plate containing 200 µL of 50 mM NaCO_3_ at pH 9.6. The plates were then stored overnight at 4°C, followed by three washes with PBS. Subsequently, the plates were incubated with DiI‐ox‐LDL at concentrations ranging from 0 to 200 µg/mL for a duration of 2 h at 37°C. After the incubation, the plates were washed three times with PBS containing 0.1% Triton‐X100. The fluorescence intensity was measured using a Biotek Synergy HT plate reader with excitation at 530/25 nm and emission at 590/35 nm.

The SPR assay utilized the dual‐flow channels of the MP‐SPR Navi 210A instrument (BioNavis Ltd., Tampere‐region, Finland) to document all measurements and kinetic parameters. The gold chips (BioNavis Ltd.) used in this assay were specifically designed based on the Kretschmann prism configuration.

For MST assay, the concentration of Dil‐ox‐LDL was constantly set to 0.05 mg/mL, and different concentrations of rhGPX4 from 2 µM to 0.122 nM were used. Nano Temper Monolith NT.115 (Nano Temper Technologies GmbH, Munich, German) was set to 60% Pico‐RED and MST 60% power. Affinity software was used to analyze the data.

### 4.10 AS model in APOE^−/−^ mice

Twenty‐four 6‐week‐old male ApoE^−/−^ mice were purchased from the Model Animal Research Center of Nanjing University. The Institutional Animal Care and Utilization Committee of Fudan University Pudong Animal Experimental Center granted approval for all specific experimental procedures (2019‐M‐02). All the mice (six per group) were equally and randomly divided into the normal, HFD, HFD + ferrostatin‐1, and HFD + liproxstatin‐1 groups. The normal group was feed with normal diet, all other groups were fed with HFD (Research Diets, Inc. New Brunswick, NJ, USA) for 3 months. Accompanied with HFD, mice in ferrostatin‐1 group were injected (i.p.) with 1 mg/kg ferrostatin‐1 (diluted in 10% DMSO, 40% PEG300, 5% Tween‐80, and 45% saline) every 2 days. Mice in liproxstatin‐1 group were injected (i.p.) with 10 mg/kg liproxstain‐1 (diluted in similar solvent of ferrostatin‐1 group) every 2 days. Normal group and HFD group both received injection of blank solvent every 2 days.

After 3 months, all the mice were anaesthetized with 1% sodium pentobarbital and drawn blood from heart. Heart, liver, and aorta were harvested and fixed with 4% paraformaldehyde. Oil Red O staining for aorta and aortic sinus was performed as described above. Masson trichrome staining, TEM, IHC, and measurement of lipids were performed as described in previous research.^44^, ^45^ NAS score was analyzed based on hematoxylin and eosin staining of liver. NAS include individual biopsy scores for steatosis (0−3), lobular inflammation (0−3), and hepatocellular ballooning (0−2) (Table S2).^46^ Serum ox‐LDL of mice was detected by commercial ELISA assay kit (Cat. CSB‐E07933m; CUSBIO, Wuhan, China). Methods for serum lipidome analysis and gut flora analysis were shown in supplementary files.

### 4.11 Serum of community screening population and plaque of carotid artery AS patients

We obtained the serum and clinical features of the newly collected (from December 2019 to December 2020) community screening population from the Pudong New Area Stroke Screening Project, including 57 cases of normal people and 111 cases of AS patients diagnosed as carotid plaque by carotid artery ultrasound. The specimen of carotid plaque was acquired from a 56 years old male patient undergoing CEA at Shanghai Pudong Hospital. Serum ox‐LDL was detected by commercial ELISA assay kit (Cat. 10‐1143‐01; Mercodia, Uppsala, Sweden). The Ethical Committee of Shanghai Pudong Hospital approved this research. All participants gave their written informed consent.

### 4.12 IHC staining of GPX4 in carotid artery AS patients

The carotid plaque specimens were obtained from 50 patients who underwent CEA at Shanghai Pudong Hospital between August 2019 and July 2021 (Table S3). The patients included in the study met specific criteria: carotid stenosis of 50−99% with a history of stroke or transient ischemic attack (TIA) symptoms within the past 6 months, or carotid stenosis of 60−99% without stroke but with TIA symptoms within the past 6 months. The research was approved by the Ethical Committee of Shanghai Pudong Hospital, and all participants provided written informed consent prior to the surgery. Furthermore, the patients were categorized into stable and vulnerable groups based on predefined criteria from previous studies.^45^ IHC was performed as previous report using anti‐GPX4 (1:100; Abclonal Technology, Inc., Wuhan, China) rabbit polyclonal antibodies.^43^

### 4.13 Statistical analysis

The statistical analysis of all experimental data was conducted using IBM SPSS 19.0 software. The data were presented as mean ± sd. To visualize the statistical results, Graphpad Prism version 7.0 software was utilized. For comparison between two groups, the *T*‐test was employed. One‐way ANOVA analysis was used to compare multiple groups, while pairwise comparison within the group was performed using the LSD‐*t* test. In cases where continuous variables did not follow a normal distribution, the Wilcoxon rank sum test was applied, particularly in the analysis of community screening population. A significance level of *p* < 0.05 was considered statistically significant.

## AUTHOR CONTRIBUTIONS

Zhou Yang and Jinyun Tan contributed to the manuscript writing and data processing. Dejun Wu and Weihao Shi contributed to specimens’ collection. Ping Liu and Yue He contributed to the construction of mice model. Rui Wang contributed to the statistical analysis. Bo Yu contributed to the design of the study. All authors read and approved the final version of the manuscript.

## CONFLICT OF INTEREST STATEMENT

The authors declare that there is no conflict of interest that could be perceived as prejudicing the impartiality of the research reported.

## PATIENT CONSENT FOR PUBLICATION

Written informed consent for publication was obtained from all the participants.

## ETHICS STATEMENT

All procedures involving human participants were performed in accordance with Shanghai Pudong Hospital ethical committee (2017‐QWJW‐010) and with the 1964 Declaration of Helsinki and its later amendments or comparable ethical standards. All patients provided their written informed consent. The Institutional Animal Care and Utilization Committee of Fudan University Pudong Animal Experimental Center granted approval for all animal experimental procedures (2019‐M‐02).

## Supporting information

Supporting information

Supporting information

## Data Availability

The datasets used and analyzed during the current study are available from the corresponding author on reasonable request.
