# Supplementary material for: Antiferroptosis therapy alleviated the development of atherosclerosis
Source: MedComm (2020). 2024 Apr 4;5(4):e520. doi: 10.1002/mco2.520 (PMC10993356; doi:10.1002/mco2.520)
Supplement: Supplementary file 1 — Supporting information [file MCO2-5-e520-s001.docx]

Supplementary files for

**Anti-ferroptosis therapy alleviated the development of atherosclerosis**

Zhou Yang^1, 2, 3, 4#^, Yue He^5, 6, 7#^, Dejun Wu^8#^, Weihao Shi^9^, Ping Liu^5, 10*^, Jinyun Tan^9*^, Rui Wang^11*^, Bo Yu^1, 2, 3, 9*^

^1^Department of Vascular Surgery, Shanghai Pudong Hospital, Fudan University Pudong Medical Center, Shanghai 201399, China.

^2^Fudan Zhangjiang Institute, Fudan University, Shanghai 201203, China.

^3^Shanghai Key Laboratory of Vascular Lesions Regulation and Remodeling, Shanghai 201399, China.

^4^Department of Head and Neck Surgery, Fudan University Shanghai Cancer Center, Shanghai 200032, China

^5^Shanghai University of Traditional Chinese Medicine, Shanghai, China.

^6^Department of Cardiology, Shanghai Eighth People's Hospital, Shanghai, China

^7^Shanghai Engineering Research Center of AⅠ Technology for Cardiopulmonary Diseases, Shanghai, China

^8^Department of General Surgery, Shanghai Pudong Hospital, Fudan University Pudong Medical Center, Shanghai 201399, China.

^9^Department of Vascular Surgery, Huashan Hospital Affiliated to Fudan University, Shanghai 200040, China.

^10^Shanghai University of Traditional Chinese Medicine, Department of Cardiology, Longhua Hospital, Shanghai 200032, China

^11^Department of Cardiovascular Surgery, Nanjing First Hospital, Nanjing Medical University, Nanjing, China.

^#^Contributed equally to this work.

^*^Co-corresponding authors:

Bo Yu, Department of Vascular Surgery, Shanghai Pudong Hospital, Fudan University Pudong Medical Center, 2800 Gongwei Road, Shanghai, 201399, China; Email: paul.yubo@gmail.com

Rui Wang, Department of Cardiovascular Surgery, Nanjing First Hospital, Nanjing Medical University, Nanjing, 68 Changle Road, Nanjing 210006, China. Email: [wr1582@njmu.edu.cn](mailto:wr1582@njmu.edu.cn)

Jinyun Tan, Department of Vascular Surgery, Huashan Hospital Affiliated to Fudan University, Shanghai 200040, China. Email: [m.tan@fudan.edu.cn](mailto:m.tan@fudan.edu.cn)

Ping Liu, Shanghai University of Traditional Chinese Medicine, Department of Cardiology, Longhua Hospital, Shanghai, China, E-mail: liuping0207@126.com

**Running Title:** Anti-ferroptosis treatment in atherosclerosis

**File list:**

1. Table S1
2. Table S2
3. Figure S1
4. Figure S2
5. Figure S3

**Supplementary material and methods**

***Serum lipidome analysis***

The lipids were extracted using the Methyl-tert-Butyl Ether (MTBE) method through serum^1^. For LC separation, reverse phase chromatography was employed with a CSH C18 column. To dissolve the lipid extracts, 200μL of 90% isopropanol/acetonitrile was used, followed by centrifugation at 14000 g for 15 minutes. 3 μL sample was then injected. Mass spectra were obtained using the Q-Exactive Plus in both positive and negative modes. The ESI parameters were optimized and preset for all measurements, including a source temperature of 300 °C, capillary temperature of 350 °C, ion spray voltage of 3000V, S-Lens RF Level of 50%, and a scan range of m/z 200–1800 for the instruments.

“Lipid Search” is an MS/MS-based search engine designed for the identification of lipid species. It encompasses over 30 lipid classes and a vast database of more than 1,500,000 fragment ions^2^. The mass tolerance for both precursor and fragment ions has been set at 5 ppm.

***Gut flora analysis***

The E.Z.N.A.® soil DNA Kit (Omega Bio-tek, Norcross, GA, U.S.) was utilized to extract total DNA from feces samples, following the manufacturer's protocol. Subsequently, all DNA samples underwent quality checks and their concentration was determined using NanoDrop 2000 spectrophotometers (Thermo Fisher Scientific, Wilmington, DE, USA). Bacterial 16S rRNA gene fragments (V3-V4) were then amplified from the extracted DNA using primers 338F (5'-ACTCCTACGGGAGGCAGCAG-3') and 806R(5'-GGACTACHVGGGTWTCTAAT-3'), with the following PCR conditions: 30 s at 95 °C, 30 s at 55 °C, and 45 s at 72 °C for 27 cycles. Finally, the amplicons were subjected to paired-end sequencing on the Illumina MiSeq sequencing platform using PE300 chemical at Majorbio Bio-Pharm Technology Co. Ltd. (Shanghai, China).

Following the demultiplexing process, the resulting sequences underwent merging using FLASH (v1.2.11) and were then subjected to quality filtering using fastp (0.19.6). Subsequently, the high-quality sequences were de-noised utilizing the DADA2 plugin within the Qiime2 (version 2020.2) pipeline, employing recommended parameters. This approach enables the attainment of single nucleotide resolution by leveraging error profiles present within the samples. The de-noised sequences obtained through DADA2 are commonly referred to as amplicon sequence variants (ASVs). To mitigate the potential impact of sequencing depth on alpha and beta diversity measurements, the number of sequences from each sample was rarefied to 4000. Despite this reduction, an average Good's coverage of 97.90% was still achieved. Taxonomic assignment of the ASVs was carried out using the Naive Bayes consensus taxonomy classifier, which is implemented in Qiime2 and utilizes the SILVA 16S rRNA database (v138).

| **Gene** | **Forward Primer** | **Reverse Primer** |
| --- | --- | --- |
| **ICAM-1** | ATGCCCAGACATCTGTGTCC | GGGGTCTCTATGCCCAACAA |
| **VCAM-1** | GGGAAGATGGTCGTGATCCTT | TCTGGGGTGGTCTCGATTTTA |
| **IL1B** | ATGATGGCTTATTACAGTGGCAA | GTCGGAGATTCGTAGCTGGA |
| **TNFA** | CCTCTCTCTAATCAGCCCTCTG | GAGGACCTGGGAGTAGATGAG |
| **GPX4** | GAGGCAAGACCGAAGTAAACTAC | CCGAACTGGTTACACGGGAA |
| **Actin** | GGGACCTGACTGACTACCTC | TCATACTCCTGCTTGCTGAT |

Table S1. The Primers of RT-qPCR

Table S2. NAS score system

| Histological features | Area | Score |
| --- | --- | --- |
| Steatosis | < 5% | 0 |
|  | 5–33% | 1 |
|  | 33–66% | 2 |
|  | > 66% | 3 |
| Lobular inflammation | None | 0 |
|  | < 2 foci per 200X field | 1 |
|  | 2–4 foci per 200X field | 2 |
|  | > 4 foci per 200X field | 3 |
| Ballooning degeneration | None | 0 |
|  | A few | 1 |
|  | Present in many cells | 2 |


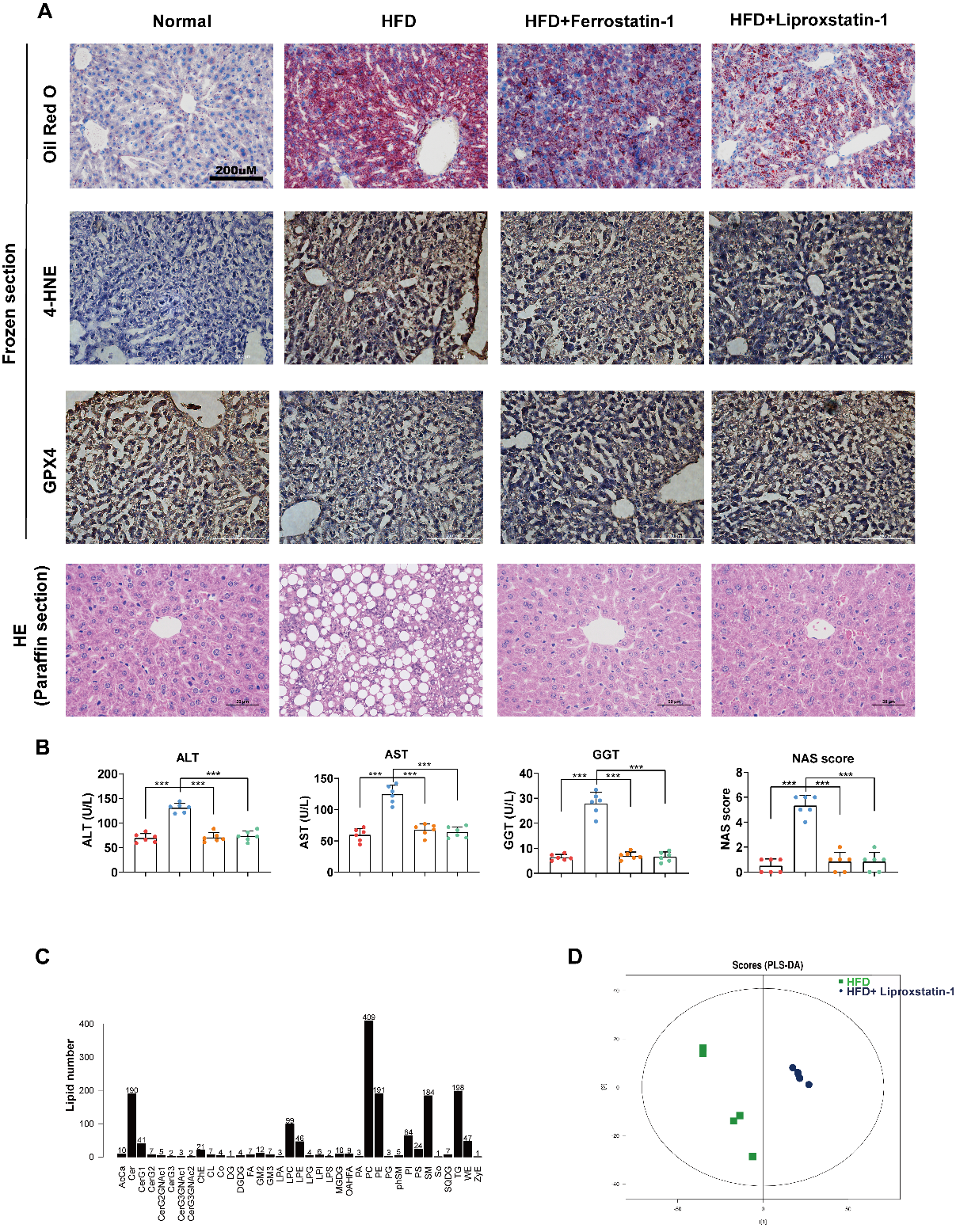


Figure S1. A. Lipid accumulation, expression of 4-HNE and GPX4 in liver of mice. B. Serum ALT, AST, GGT and non-alcoholic fatty liver disease activity (NAS) score in each group. C. Lipids number in serum of mice detected by LC/MS. D. PLS-DA analysis performed to screen different lipids between groups. (*P<0.05, **P<0.01, ***P<0.001).


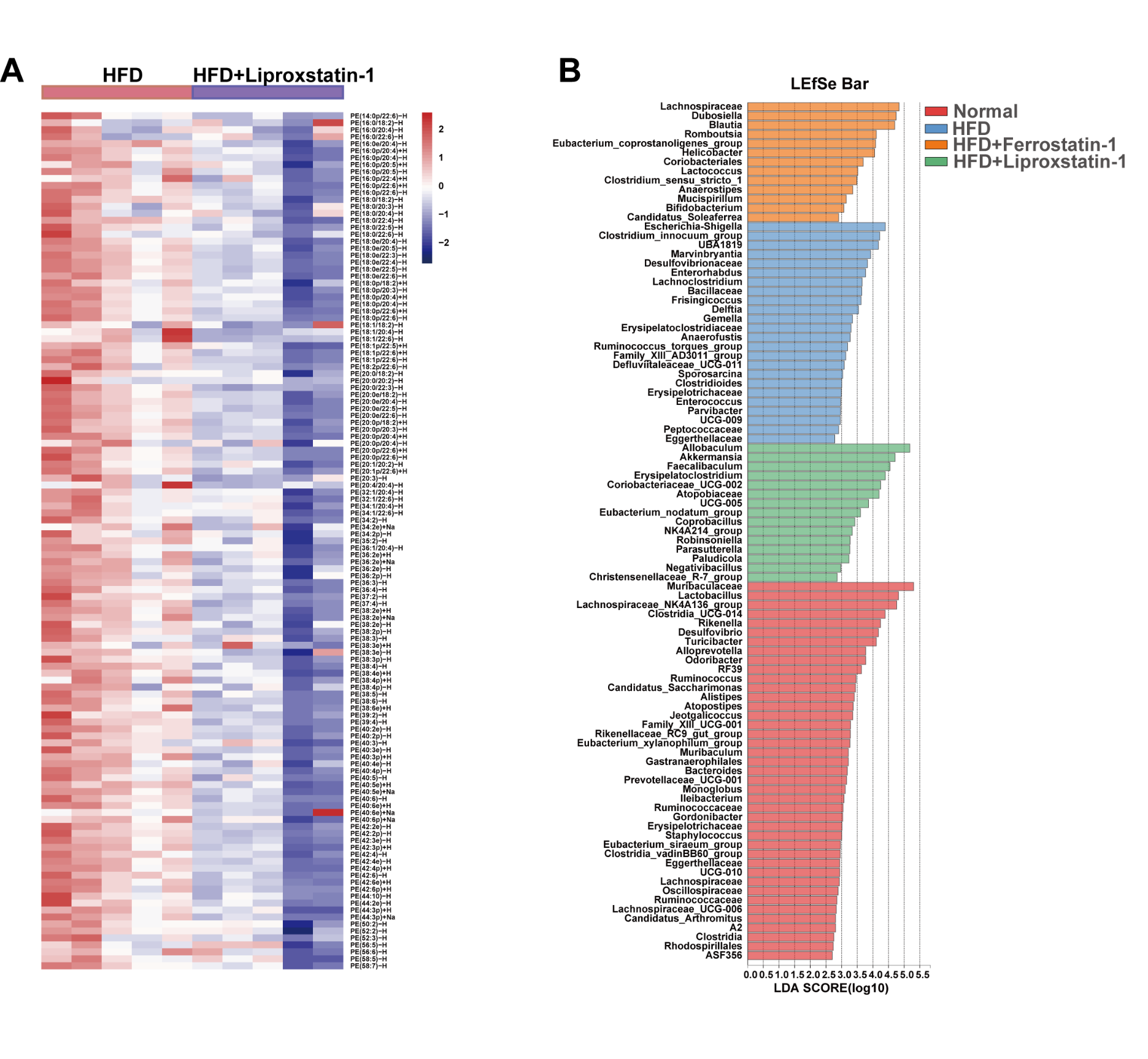


Figure S2. Liproxstatin-1 repressed various PUFA-PEs in mouse serum.


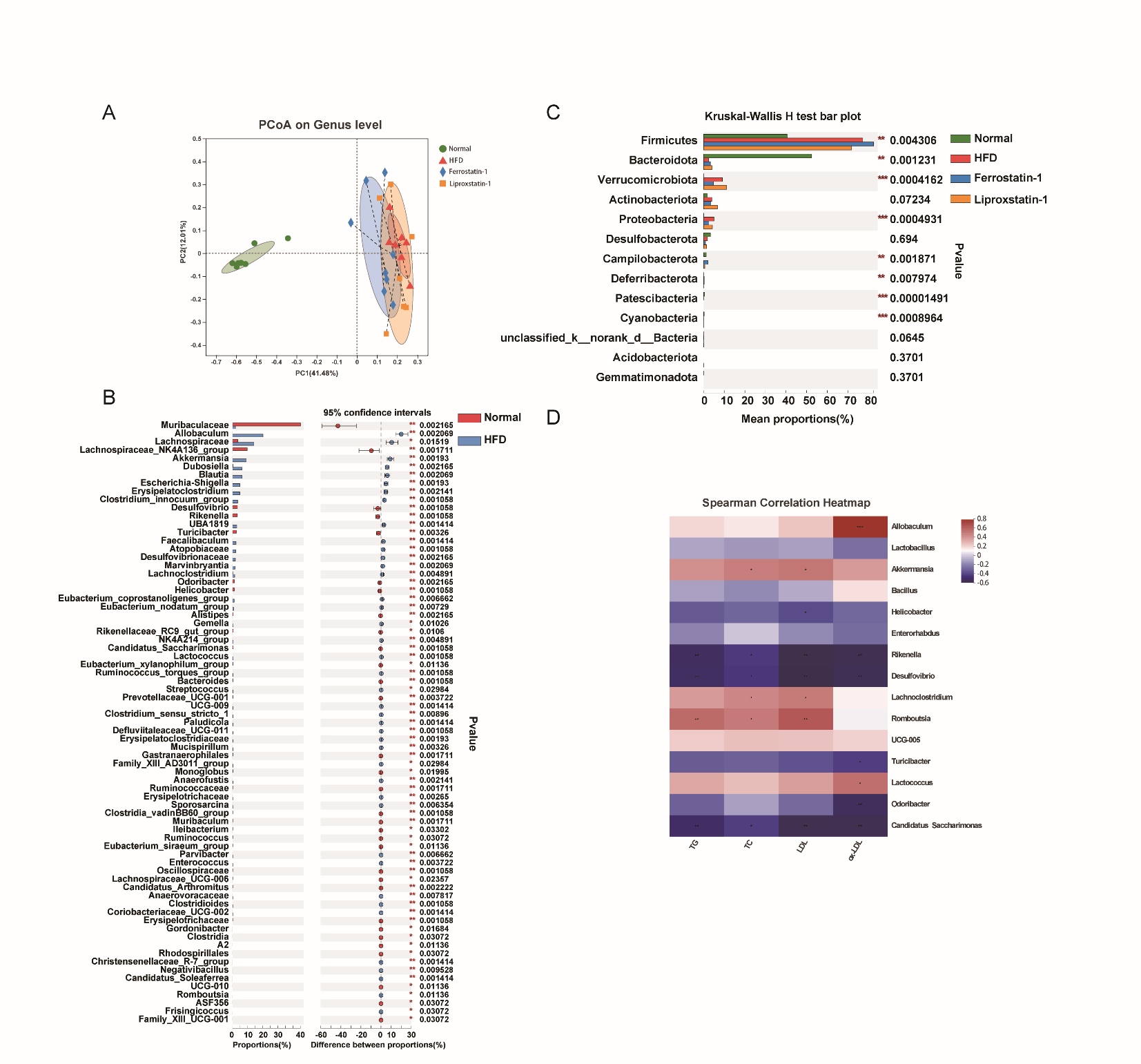


Figure S3. A. PCoA analysis of of the gut microbial community in each group. B. Gut microbiota with a significant difference between the Normal group and HFD group at the genus level by the Wilcoxon test. C. Comparison of the gut microbiota of the Normal, HFD, Ferrostatin-1 and Liproxstatin-1 groups at the phylum level by the Kruskal-Wallis test. D. Correlation between blood lipids and gut microbiota. (*P<0.05, **P<0.01, ***P<0.001).

**References**

1. Eggers LF, Schwudke D. Lipid Extraction: Basics of the Methyl-tert-Butyl Ether Extraction. In: Wenk MR, ed. *Encyclopedia of Lipidomics*. Springer Netherlands; 2016:1-3.

2. Taguchi R, Ishikawa M. Precise and global identification of phospholipid molecular species by an Orbitrap mass spectrometer and automated search engine Lipid Search. *Journal of chromatography A*. Jun 18 2010;1217(25):4229-39. doi:10.1016/j.chroma.2010.04.034
